# Type I Collagen and Strontium-Containing Mesoporous Glass Particles as Hybrid Material for 3D Printing of Bone-Like Materials

**DOI:** 10.3390/ma11050700

**Published:** 2018-04-28

**Authors:** Giorgia Montalbano, Sonia Fiorilli, Andrea Caneschi, Chiara Vitale-Brovarone

**Affiliations:** 1 Politecnico di Torino, Department of Applied Science and Technology, Corso Duca degli Abruzzi 24, 10129 Torino, Italy; giorgia.montalbano@polito.it (G.M.); sonia.fiorilli@polito.it (S.F.); 2 DIEF—Department of Industrial Engineering and RU INSTM, Università degli Studi di Firenze, Via S. Marta 3, 50139 Firenze, Italy; andrea.caneschi@unifi.it

**Keywords:** type I collagen, mesoporous bioactive glasses, bone tissue engineering, bioactive materials, hybrid systems, nanostructured materials, biomimicry

## Abstract

Bone tissue engineering offers an alternative promising solution to treat a large number of bone injuries with special focus on pathological conditions, such as osteoporosis. In this scenario, the bone tissue regeneration may be promoted using bioactive and biomimetic materials able to direct cell response, while the desired scaffold architecture can be tailored by means of 3D printing technologies. In this context, our study aimed to develop a hybrid bioactive material suitable for 3D printing of scaffolds mimicking the natural composition and structure of healthy bone. Type I collagen and strontium-containing mesoporous bioactive glasses were combined to obtain suspensions able to perform a sol-gel transition under physiological conditions. Field emission scanning electron microscopy (FESEM) analyses confirmed the formation of fibrous nanostructures homogeneously embedding inorganic particles, whereas bioactivity studies demonstrated the large calcium phosphate deposition. The high-water content promoted the strontium ion release from the embedded glass particles, potentially enhancing the osteogenic behaviour of the composite. Furthermore, the suspension printability was assessed by means of rheological studies and preliminary extrusion tests, showing shear thinning and fast material recovery upon deposition. In conclusion, the reported results suggest that promising hybrid systems suitable for 3D printing of bioactive scaffolds for bone tissue engineering have been developed.

## 1. Introduction

The relevant clinical problem represented by the significant number of bone defects and bone injuries has led to an increasing demand for new biomaterials suitable for bone regeneration and replacement, especially for fractures arising from specific pathological conditions, such as osteoporosis [1,2,3,4]. Considering that all the current methods have shown significant drawbacks, tissue engineering holds potential in providing an improved clinical therapy, as an alternative viable solution to repair bone injuries [2,4].

In this frame, biomimetic and bioactive materials are emerging as promising systems able to direct and modulate cell behaviour by providing specific instructive signals, leading to the potential regeneration of healthy tissues [3,5]. These types of materials are often used for the design of scaffolds that aim to mimic structural, mechanical, and biological properties of natural tissues, additionally considering the complex structure at the nanoscale [4].

Bone is a highly complex structure, hierarchically organised, and it is mainly based on assembled type I collagen fibrils that offer an organic substrate suitable for hydroxyapatite (HA) crystal precipitation [6,7].

Type I collagen represents the most abundant structural protein of the human body and the main organic component of the natural bone tissue (about 90% of the protein content), playing a fundamental role in its mineralization [4,8]. Moreover, this structural protein is well known to influence different aspects of cell behaviour, like cell-adhesion, proliferation, and differentiation thanks to the high density of the Arg-Gly-Asp (RGD) and other functional aminoacidic sequences [7,9]. However, the lack of biomechanical stiffness and the rapid biodegradation often require the stabilisation of collagen-based biomaterials that can be achieved with chemical crosslinking methods or by adding components with higher mechanical properties [6,8,10,11].

Type I collagen is, therefore, often combined with inorganic phases, such as calcium phosphates or bioactive glasses, to obtain biomimetic composites for bone tissue engineering [12,13].

In the literature, different examples of collagen-based constructs combined with bioactive glasses have been proposed for bone tissue engineering applications, such as freeze-dried scaffolds incorporating bioactive glass nanofibers [6,14], injectable collagen-bioactive glass hybrid systems [1], or bioactive glass surface coating of collagenous matrices [12,15].

The combination of bioactive glasses with type I collagen has frequently been reported to have excellent biocompatibility and adequate biomechanical properties [6]. The well-known bioactive properties are even more enhanced in mesoporous bioactive glasses (MBGs) due to their higher surface area and reactivity [10,16].

Different studies have proved that bioactive glasses and MBGs are able to enhance bone formation and integration with the surrounding bone tissue in vivo [12]. Furthermore, they can act as mineralization agents in composite materials, increasing the ability of collagenous matrices to promote nucleation and subsequent growth of calcium phosphates, as well as improving the structural properties of collagen constructs [1,4,6,12,14,17]. With the aim to increase the osteogenic properties of the material, strontium ions can be incorporated in the glass composition. In fact, strontium ions have proved to stimulate osteoblast activity and, thus, increase bone formation, while reducing bone resorption [16,18,19,20,21,22,23].

The final features of each construct depend not only on the chosen materials, but also on the used fabrication technique. Different approaches have been studied to produce nanostructured bone scaffolds, such as electrospinning [24,25,26], thermally-induced phase separation, molecular self-assembling [27], 3D printing, or combinations of them [5,12,13]. However, although all the reported technologies are able to create nanostructured constructs combining different types of both synthetic and natural biomaterials, most of them result in a lack of control over the scaffold porosity and structure. Among nano- and micro-fabrication technologies, 3D printing is the only technique able to step over the structure limitation while offering high biomimicry and a reproducible, versatile process [4,5,13,28,29,30]. However, the printing of highly-complex nanostructures requires the use of biomaterials characterized by specific properties, such as shear thinning to enable material extrusion through very thin needles, as well as yield stress and fast recovery after deposition to improve the printing fidelity [28,29,31,32].

In this context, the present study, framed in the ERC BOOST project, aimed to design and characterise a bioactive formulation based on the combination of type I collagen and mesoporous bioactive glass particles containing strontium ions, suitable for the 3D printing of nanostructured scaffolds mimicking the structure of the natural bone tissue. Since most of the biomaterials currently optimised for 3D printing applications are based on synthetic polymers, due to their more versatile properties, the development and the optimization of a collagen-based system adds relevance to the present study [28,29,32]. In addition, although a significant number of hybrid systems based on the combination of an inorganic phase and collagen has already been reported, the designed formulation involving Sr-containing mesoporous bioactive glasses (MBGs), to the best of the authors’ knowledge, has not yet been reported in the literature [12].

With the aim to investigate the physico-chemical and the nanostructural features of the obtained composite, the sol-gel transition of the collagenous suspension at 37 °C was exploited to obtain bulk samples, subsequently freeze-dried and characterized. The potential bioactivity was studied by detecting the deposition of HA crystals and was then confirmed by X-ray diffraction (XRD) and field emission scanning electron microscopy (FESEM) analyses. The water uptake ability and the release of Sr^2+^ species were also analysed.

Finally, the suitability of collagen/Sr-containing MBG suspensions for 3D printing applications was assessed by rheological analyses, particularly investigating the suspension viscosity at different shear stress conditions and the visco-elastic properties of the material after gelation. To complement the rheological study, preliminary manual extrusion tests were conducted by using syringes equipped with 32 G needles in order to investigate the eventual needle clogging induced by the needle size. The deposited strands were analysed to detect any variation in the final nanostructure induced by the material processing.

## 2. Materials and Methods

### 2.1. Preparation of Sr-Containing MBGs by the Aerosol Spray-Assisted Approach

Mesoporous bioactive glass microspheres with 4% molar percentage of strontium (Sr/Ca/Si = 4/11/85) were synthesised according to the protocol previously reported by Molino et al. [33] and are hereinafter mentioned as MBG_Sr4%. In brief, a pre-hydrolysed TEOS solution was added drop by drop to an aqueous solution of Pluronic P123 (EO_20_PO_70_EO_20_, average M_n_ ~5800, Sigma Aldrich, Milan, Italy) and kept stirring for about 1 h. The calculated amount of calcium nitrate tetrahydrate (Ca(NO_3_)_2_ 4H_2_O, 99%, Sigma Aldrich, Milan, Italy) and strontium chloride (SrCl_2_, Purity, Sigma Aldrich, Milan, Italy)were subsequently added and the final solution was sprayed (Büchi, Mini Spray-Dryer B-290, Büchi Labortechnik AG, Flawil, Switzerland) using nitrogen as the atomizing gas. The resulting powders were collected and calcined at 600 °C in air for 5 h using a furnace (Carbolite 1300 CWF 15/5, Carbolite LTD, Hope Valley, UK).

### 2.2. Preparation of Collagen/MBG_Sr4% Suspensions

The weight amounts of collagen powders and MBG particles were calculated in order to reproduce the volume percentages of the organic and inorganic phases in the natural bone tissue (53 vol % of collagen and 47 vol % of the inorganic phase) [34,35,36]. According to the manufacturer’s instructions, collagen powders (Type I collagen from bovine Achilles tendon, Vornia Biomaterials, Dublin, Ireland) were dissolved in 0.5 M acetic acid solution under stirring at 4 °C, until complete dissolution. MBG_Sr4% particles were dispersed in 0.5 M acetic acid by sonication for 5 min using an ultrasonic bath (Digitec DT 103H, Bandelin, Berlin, Germany) and subsequently added dropwise to the collagen solution, achieving the designed ratio of collagen and the inorganic phase. The resulting collagen/MBG_Sr4% suspension was mixed for about one hour in order to ensure optimal homogeneity. In order to promote the sol-gel transition and the collagen fibre reconstitution, the acidic pH was neutralised by adding 1 M NaOH solution reaching a final collagen concentration of about 1 wt % [8]. The entire process was performed at 4 °C in order to avoid premature collagen cross-linking.

### 2.3. Preparation of Collagen/MBG_Sr4% Bulk Samples

Bulk samples were prepared to investigate the physico-chemical properties of the composite material in the solid form. In details, 350 μL of the collagen/MBG_Sr4% suspension were pipetted in a silicon mould creating samples with 10 mm diameter and 5 mm thickness. The mould was then left at 37 °C for 3 h in order to allow for the sol-gel transition resulting from the natural physical crosslinking of collagen molecules. The solid samples were then collected and stored at 4 °C until testing.

### 2.4. Micro-Nano Structure Evaluation by Field Emission Scanning Electron Microscopy (FESEM)

Immediately after preparation, collagen/MBG_Sr4% samples were incubated in Dulbecco phosphate buffered saline (D-PBS, Sigma Aldrich, Milan, Italy) at 37 °C for 24 h and subsequently frozen at −20 °C. The frozen samples were then lyophilised for 48 h in an Alpha 2-4 LD freeze-dryer (Martin Christ, Osterode am Harz, Germany) under vacuum (<0.1 mbar). In order to observe the collagen fibre reconstitution and the inorganic particle distribution, cross-sections of lyophilised samples were chromium-coated to enhance sample conductivity and analysed by field-emission scanning electron microscopy (FESEM) using a ZEISS MERLIN (ZEISS MERLIN Instrument, Oberkochen, Germany) instrument.

### 2.5. Collagen/MBG_Sr4% Construct Bioactivity

The bioactivity of collagen/MBG_Sr4% samples was investigated by soaking each sample in 3 mL of simulated body fluid (SBF), following a similar procedure to the one reported for bioactivity studies on composites of collagen and bioactive glass particles [9,10,14,37]. After immersion in SBF, the samples were incubated at 37 °C for up to seven days. At each time point (3 h, 1 day, 3 days, and 7 days) the samples were rinsed in distilled water twice and collected for further analyses. Three samples were tested for each time point. To evaluate the HA crystal formation by FESEM, ATR-FTIR, and XRD analyses, the collected samples were frozen at −20 °C and subsequently lyophilised for 48 h as previously reported.

X-ray diffraction analyses on collagen/MBG_Sr4% samples were conducted by using a Bruker New D8 Da Vinci diffractometer (Cu−Kα radiation, 40 kV × 40 mA, Bruker, Billerica, MA, USA), equipped with a Bruker LYNXEYE-XE detector, a scanning range of 2θ = 10−80°, 0.013° increments of 2θ, and a counting time of 0.5 s/step. The acquired graphs were compared with the one of HA as a reference using the peaks identified by the International Centre for Diffraction Data.

FTIR spectra, in the 4000–650 cm^−1^ range, were collected by using a Bruker Equinox 55 spectrometer (Bruker, Billerica, MA, USA), equipped with MCT cryodetector, at a spectral resolution of 4 cm^−1^ and the accumulation of 32 scans, by using the attenuated total reflection (ATR) mode.

Finally, the HA particle size after seven days of incubation was calculated by means of the Scherrer equation, considering the half-width of the peak at 2θ = 26°.

### 2.6. Water Uptake Ability of Collagen/MBG_Sr4% Constructs

Collagen/MBG_Sr4% samples were lyophilised immediately after preparation and weighed (*Wd*) before performing the test. Subsequently, each sample was immersed in 3 mL of deionised water and incubated for 3, 24, and 48 h at 37 °C. At each time point the samples were weighed after removing the excess of water to obtain the swollen sample weight (*Ww*). The resulting percentage of the swelling ratio was calculated according to the following equation:

Swelling ratio (%) = 100 × [(*Ww* − *Wd*)/*Wd*]

Three samples were considered for each time point and the results were reported as the mean ± standard deviation.

### 2.7. Evaluation of Sr Ion Release from Collagen/MBG_Sr4% Composites

The pattern of Sr ion release from collagen/MBG_Sr4% samples was assessed following incubation in 3 mL of Tris HCl buffer (Tris(hydroxymethyl)aminomethane (Trizma) (Sigma Aldrich, Milan, Italy) 0.1 M, pH 7.4) at 37 °C. At predefined time steps (24 h, 3 days, 7 days, 14 days) half of the supernatant was collected and replaced by the same amount of fresh buffer previously equilibrated at 37 °C. To measure the concentration of released Sr ions, the collected supernatant was analysed by the inductively-coupled plasma atomic emission spectrometry technique (ICP-AES) (ICP-MS, Thermoscientific, ICAP Q, Waltham, MA, USA) after proper dilutions. Analyses were conducted in triplicate and the results are reported as the mean ± standard deviation.

### 2.8. Rheology

The rheological analyses were conducted by using a DHR-2 controlled stress rotational rheometer (TA Instruments, Waters, New Castle, DE, USA). All the tests were performed using a parallel plate geometry with a diameter of 20 mm. The range of temperatures considered for the different tests ranged between 4 °C and 37 °C and was constantly controlled by a Peltier plate system. In addition, a solvent trap system was used for all the analyses performed at 37 °C in order to avoid both the solvent evaporation and the sample drying. Before starting the analyses, each sample was placed on the Peltier plate and the test geometry was set to the desired gap (between 500 and 1000 μm), removing the excess material. Each sample was used for only one test.

#### 2.8.1. Analyses on Collagen/MBG_Sr4% Suspensions

A peak hold test was performed at 4 °C to simulate the printing process by setting a shear stress value of 200 s^−1^ (100 s) and 0.1 s^−1^ (300 s) to reproduce the extrusion and the deposition step respectively. Variations in the suspension viscosity with different shear stresses (0.01–200 s^−1^) and the yield stress values were detected by flow ramps at 4 °C. Finally, a time sweep analysis was performed at 37 °C under 5% strain for 60 min to observe the sol-gel transition of the collagen/MBG systems. All the tests were performed at 4 °C to prevent material gelation.

#### 2.8.2. Analyses on Collagen/MBG_Sr4% Composites

Collagen/MBG_Sr4% suspensions were allowed to gel at 37 °C for 3 h before testing the visco-elastic properties. After sample preparation, a dynamic amplitude sweep (0.01–1% strain) analysis was conducted to investigate the increasing of storage (G’) and loss (G’’) moduli with increasing time of incubation at 37 °C and 1 Hz. The amplitude sweep test also allowed the detection of the linear visco-elastic region (LVR).

### 2.9. Preliminary Extrusion Tests with 32 G Needles

Collagen/MBG_Sr4% suspensions were extruded using a syringe equipped with 32 G needles (internal diameter of about 120 μm) to start preliminary studies on the suspension printability. The extrusion plate was kept at 37 °C in order to promote the sol-gel transition of the collagenous suspensions. Strands of extruded material were collected and prepared for FESEM analyses to investigate the material organisation at the micro- and nano-scale.

## 3. Results and Discussion

**Figure A1:**
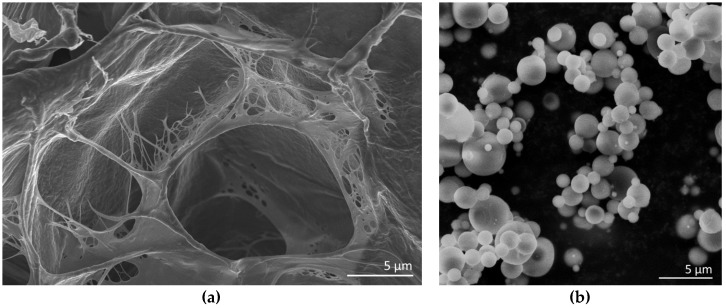
FESEM images of type I collagen lyophilised samples (**a**) and MBG_Sr4% particles (**b**).

Neutralised and properly homogenised suspensions of type I collagen and MBG_Sr4% particles were prepared and subsequently exploited to produce solid samples by promoting the sol-gel transition at 37 °C via the collagen’s physical crosslinking. MBG particles with SiO_2_-CaO composition containing 4% molar percentage of strontium were obtained by means of the aerosol spray-assisted technique. The inorganic particles showed spherical morphology with a size ranging from about 500 nm to 5 μm, specific surface area of 154 m^2^/g, and average pore diameter of about 5 nm (Figure A1) [16,38]. Both the synthesis procedure and composition of MBG_Sr particles have been optimised in previous studies, showing high Sr incorporation ability and relevant bioactive properties [33,38]. The use of spherical micro-sized particles allowed the creation of homogeneous and stable suspensions that did not show sedimentation even after one week of storage at 4 °C. Compared to micrometre-sized particles [39], recent works have proven that the use of nano-sized particles leads to improved osteoconductive properties and stiffness of the final construct due to the enhanced bioreactivity and better embedding quality [12,40]. Future works may, thus, consider the incorporation of smaller particles derived from alternative synthesis methods with the aim to improve the final properties of the hybrid system.

### 3.1. Micro-Nano Structure Evaluation by Field Emission Scanning Electron Microscopy (FESEM)

**Figure 1:**
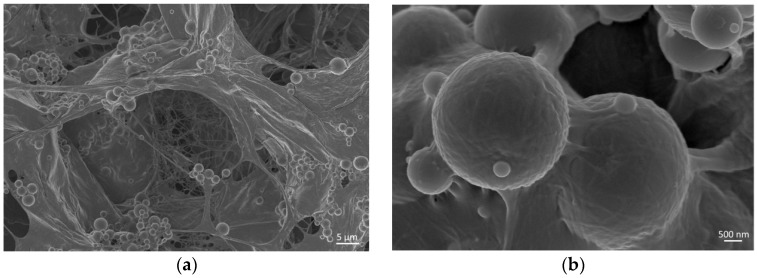
Cross-sectional FESEM images of collagen/MBG_Sr4% lyophilised samples at different magnifications: 5000× (**a**) and 50,000× (**b**).

The natural physical gelation of collagen is normally obtained by means of the neutralisation of acidic-solubilised solutions of collagen and the subsequent incubation at 37 °C, thus reproducing similar physiological conditions, where collagen molecules can organise and assemble into a gel matrix containing a large amount of water [41]. However, potential interactions with other materials or particular synthesis conditions can alter or prevent the natural self-assembling of collagen molecules [10]. The micro- and nanostructure of collagen/MBG nanocomposites were, thus, investigated by field emission scanning electron microscopy (FESEM) at different magnifications (Figure 1).

**Figure 2:**
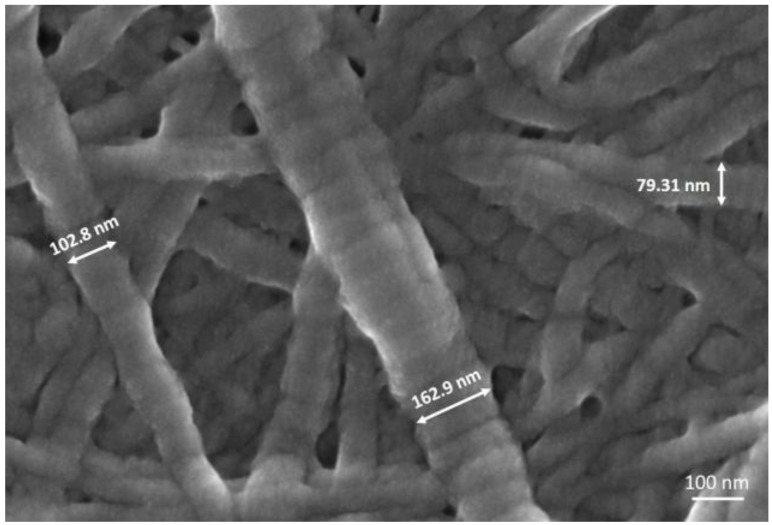
Cross-sectional FE-SEM image of lyophilized collagen/MBG_4%Sr sample at 250,000×, showing the typical banded pattern of collagen fibres.

**Figure A2:**
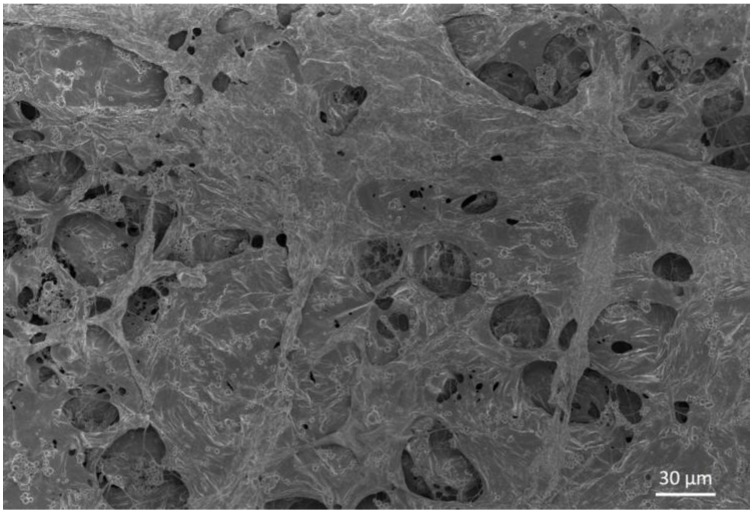
Cross-sectional FESEM image of collagen/MBG_Sr4% lyophilised samples at lower magnification (1000×). The image shows the homogeneous distribution of inorganic particles in the collagenous matrix.

FESEM images proved the successful reconstitution of collagen fibrils forming fibrous meshes and walls that properly embedded the MBG particles. Any significant agglomerate of particles was not detected confirming a good dispersion of the inorganic phase inside the collagenous matrix, also suggesting that no sedimentation processes occurred during the suspension preparation (Figure A2) [9]. The ability of collagen to organise in three-dimensional fibrous structures was further confirmed at higher magnifications (250,000×), where the collagen fibre exhibited the typical D-periodic banding pattern (D = 67 nm), due to the presence of overlapping and lacunar regions in the highly-organised supramolecular packing (Figure 2). In addition, fibre diameters detected from FESEM images, ranging from about 70 to 170 nm, were found to be consistent with data reported in the literature (50–500 nm) [7,9].

Despite the large amount of the inorganic phase in the hybrid system composition, the natural fibrillar organisation of collagen was preserved together with the homogeneous glass particle embedding, both key factors for the final design of biomimetic structures. This will allow the fabrication of hybrid 3D scaffolds able to precisely reproduce the human bone tissue ratio between collagen and the inorganic phase [34,35].

### 3.2. Collagen/MBG_Sr4% Construct Bioactivity

One of the most important aspect in bone tissue engineering is the extracellular matrix biomineralization [6,39]. The material ability to promote the deposition of bone-like apatite minerals was, thus, examined in vitro by incubating collagen/MBG_Sr4% samples in simulated body fluid (SBF) for up to seven days.

**Figure 3:**
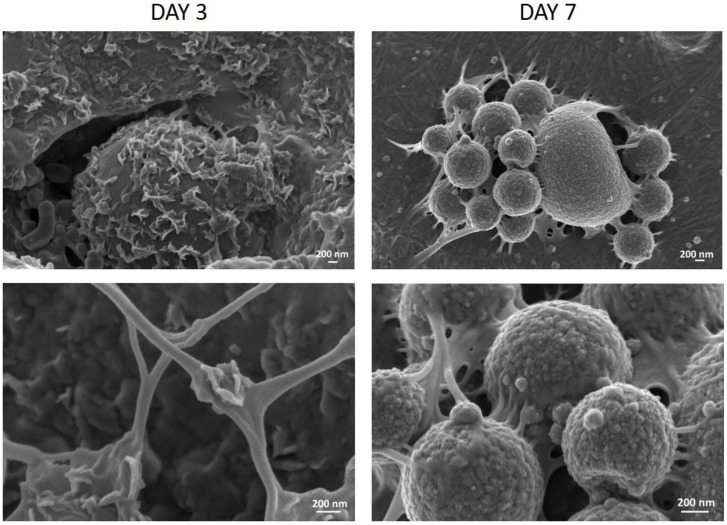
Cross-sectional FESEM images showing HA crystal deposition on collagen/MBG_Sr4% samples after three and seven days of incubation in SBF at different magnifications.

FESEM analyses revealed the formation of HA crystals homogeneously distributed on the sample cross-sections already after three days in SBF, confirming the remarkable bioactivity of the hybrid material [1,42]. Images collected after up to seven days showed a greater amount of HA deposits on both MBG particles and collagen fibres, observing a more granular shape with increasing immersion times in SBF, as shown in Figure 3.

The calcium phosphate deposition on collagen/MBG samples was further analysed by means of ATR-FTIR and XRD analyses on sample cross-sections, to confirm the chemical composition and structure of the deposits.

**Figure 4:**
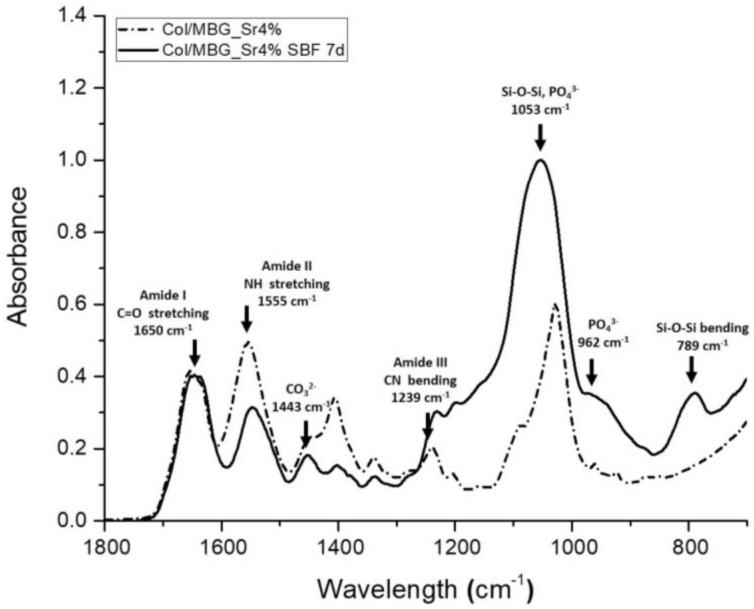
ATR-FTIR spectroscopy of collagen/MBG_Sr4% samples before and after immersion in SBF for seven days.

In the ATR-FTIR graphs the typical bands of collagen centred at 1650 cm^−1^, 1555 cm^−1^, and 1443 cm^−1^ for amide I (C=O stretching), amide II (N-H stretching), and amide III (C-N bending), respectively, were observed, while the presence of MBGs was confirmed by the Si-O-Si band at 1027 cm^–1^ [10,43,44]. As reported in Figure 4, the ATR spectra of samples incubated for seven days in SBF clearly suggest the deposition of calcium phosphates on the collagenous matrices. In detail, the phosphate deposition led to a significant increase in absorbance at 962 cm^−1^ and between 1000 and 1100 cm^−1^, where the peak at 1053 cm^−1^ resulted from both Si-O-Si and phosphate contributions. The increase of Si-O-Si absorbance was also detected at 789 cm^−1^, while carbonate formation was identified by the peak at 1443 cm^−1^ [6,39]. Similar ATR-FTIR spectra have been previously reported for bioactive glass containing collagen matrices, but after a longer time of incubation in SBF (14 days) [39]. The use of mesoporous bioactive glasses enhances the material reactivity, therefore, leading to faster deposition kinetics.

**Figure 5:**
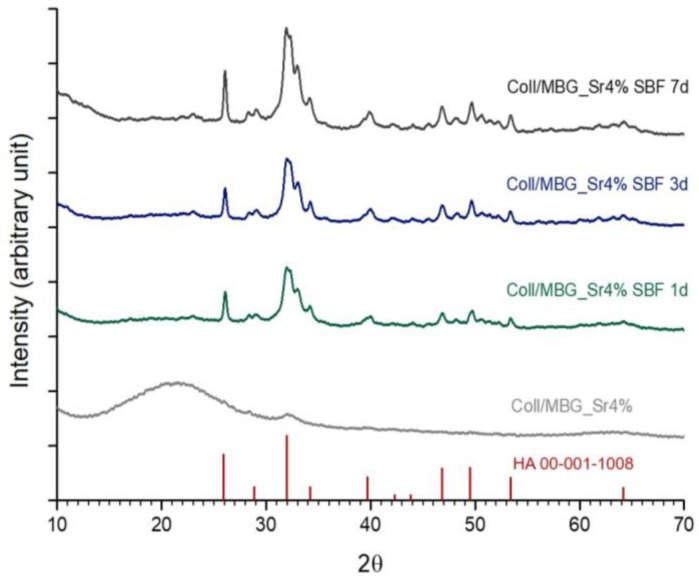
XRD of collagen/MBG_Sr4% at days 1, 3, and 7 in SBF. HA standard peaks reported by the International Centre for Diffraction Data are reported as a reference.

As reported in Figure 5, XRD analyses clearly showed the change from the broad diffraction peak corresponding to the presence of collagen and MBGs to the crystalline peaks of HA already after 24 h of incubation in SBF, even if ATR_FTIR analyses reported significant variations only at day 7. The resulting graphs showed the typical peaks of HA particularly at 26°, 28°, 31.9°, 39.6°, 46.8°, 49.3°, and 53.7°. The detected peaks have been shown to match the reference values for HA reported by the International Centre for Diffraction Data [1,37,39].

The abundant deposition of calcium phosphate crystals proves the increased bioactivity potential of the composite material due to the synergistic action of collagen and MBG, also reported in previous studies [1,37,39,45]. In fact, collagen fibres were studied to provide additional nucleation sites for HA due to the presence of acidic groups (derived from aspartic and glutamic acid) that can easily interact with calcium ions, promoting the subsequent bond with silicic acid [37]. The significant increase in absorbance of the band due to Si-O-Si groups, detected after immersion in SBF, can be ascribed to the formation of the bioactive silica layer formed by dissolution and re-precipitation reactions at the highly-reactive surface of mesoporous bioactive glasses [1,12,39].

Based on the XRD patterns of samples incubated for seven days in SBF, the Sherrer equation (calculated on the half-width of the peak at 2θ = 26°) revealed an averaged HA particle size of about 35.43 nm, similar to the values reported for natural bone crystals (50 nm) [46].

### 3.3. Water Uptake Ability of Collagen/MBG_Sr4% Constructs

Higher degrees of swelling in biomaterials normally lead to greater biocompatibility while promoting proper fluid exchange between the designed construct and the surrounding environment [47]. The water uptake ability of collagen/MBG bulk samples was studied after 3, 24, and 48 h of immersion in distilled water at 37 °C. 

**Figure 6:**
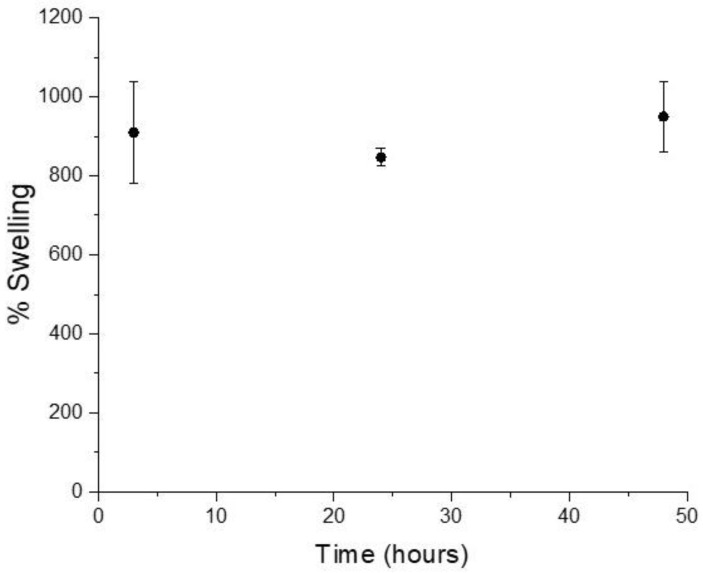
Swelling values of collagen/MBG_Sr4% samples after 3, 24, and 48 h of immersion in distilled water.

As shown in the graph (Figure 6), no significant differences were found between swelling values at different time points (909.5 ± 128%, 847.1 ± 22%, and 949.5 ± 88% after 3, 24, and 48 h, respectively), suggesting that the bulk samples already reached the equilibrium swelling after 3 h of incubation. The reported results showed the relevant ability of the collagen/MBG systems to retain water consistently with data reported for similar composite materials based on collagen and inorganic particles [47]. The high swelling capacity may be related to different factors, such as the ability to form hydrogen bonds with water molecules, as well as the material polarity. The composition of bioactive glass particles are expected to further enhance the hydrophilicity of collagenous matrices to the large amount of surface silanol groups [42].

### 3.4. Evaluation of Sr Ion Release from Collagen/MBG_Sr4% Composites

**Figure 7:**
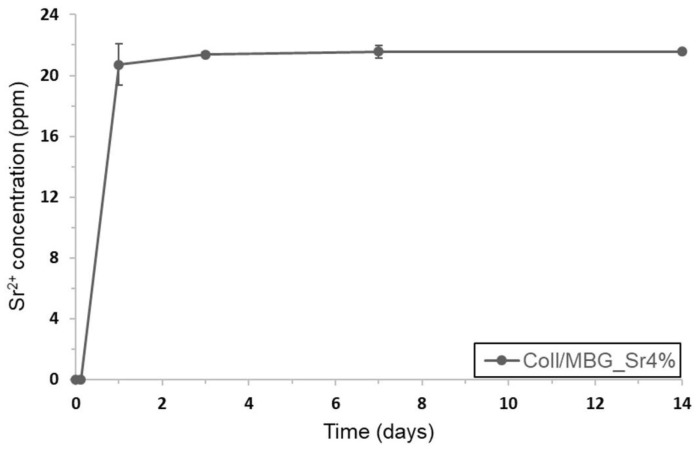
Sr^2+^ ion release from collagen/MBG_Sr4% after 3 and 24 h and 3, 7, and 14 days of immersion in Tris HCl at 37 °C.

The release of Sr^2+^ ions from MBGs embedded in the collagenous matrix was studied by soaking bulk samples in Tris-HCl at 37 °C. Figure 7 showed the ion concentration measured in the supernatant collected at different time points (3 h, 24 h, 3 days, 7 days, and 14 days).

**Figure A3:**
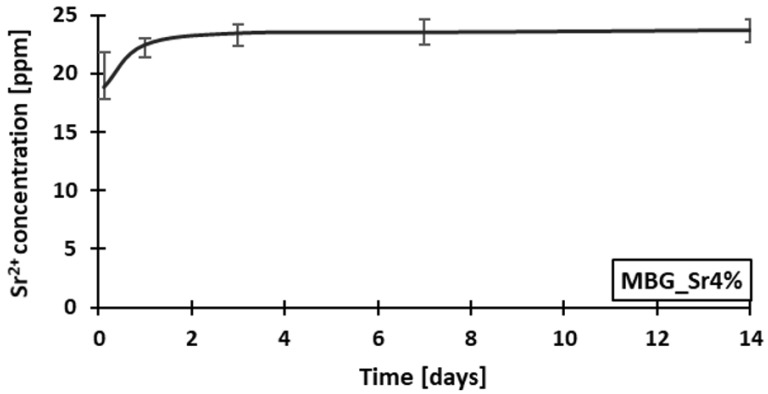
Sr ion release from MBG_Sr4% particles in Tris-HCl. The reported profile shows that all the ion content has been released after 24 h of incubation.

According to the amount of strontium initially incorporated in MBG_Sr4% and the inorganic phase content in each sample, ICP analyses proved that all the ion content was released after three days of immersion. Compared to the release data obtained from analyses on MBG_Sr4% particles alone reported by the authors in [16], the strontium ion burst release was delayed by about 24 h, whereas no ions were detected after only 3 h of sample immersion (Figure A3). The resulting data suggest that the collagenous matrix did not prevent the ion exchange between the inorganic particles and the soaking medium, but delayed the release due to the medium diffusion kinetics.

Several recent studies on Sr-containing particles have proved the significant pro-osteogenic effect induced by Sr^2+^ species released with similar concentration and kinetics [16,20,21,22]. In addition, a further control of the release kinetics could be achieved by functionalizing the external surface of the bioactive glass particles with a degradable coating.

### 3.5. Rheology

Rheological analyses are normally conducted to study the visco-elastic properties of the material by measuring the mechanical response of samples subjected to different stresses and strains. In this frame, collagen/MBG suspensions were analysed defining the variation of rheological properties with stress conditions typically occurring during the extrusion printing processes. Since the hybrid material performs a sol-gel transition at 37 °C, the influence of temperature on material properties were considered a key point in this study.

#### 3.5.1. Analyses on Collagen/MBG_Sr4% Suspensions

During the extrusion printing process, the suspension initially undergoes high shear rates (passing through the nozzle), that are immediately removed once the material has been deposited, requiring the fast recovery of the material structure (*self-healing*) [13,28,29]. A *peak hold* test was, thus, performed, observing the response of the material subjected to stress conditions similar to those occurring during the printing process. In detail, two different subsequent shear rates of 200 s^−1^ (100 s) and 0.1 s^−1^ (300 s) were applied to the collagen/MBG suspension, reproducing the extrusion and the deposition phase respectively.

**Figure 8:**
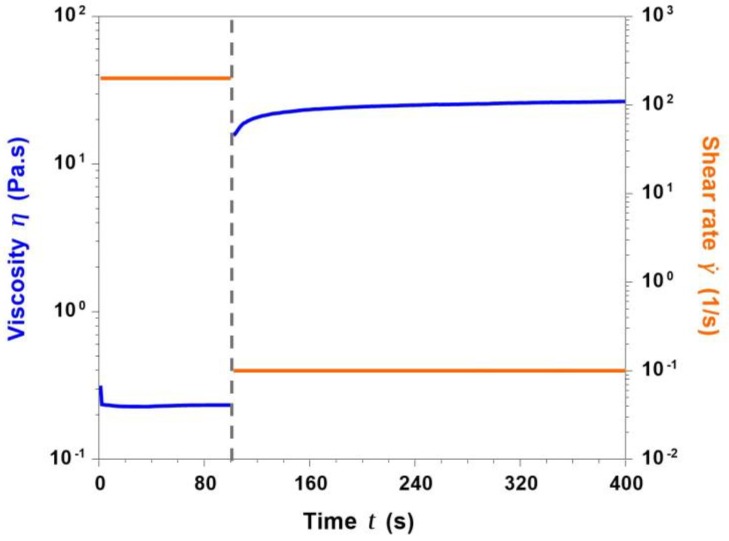
Printing process simulation by means of the peak hold test performed at 4 °C.

As shown in Figure 8, the suspension viscosity was set to very low values of about 0.23 Pa·s as a result of the application of a constant shear rate of 200 s^−1^. Once the shear stress was removed (0.1 s^−1^), viscosity sharply increased reaching plateau values of about 25 Pa·s after only 100 seconds, suggesting a fast recovery of the material molecular structure.

**Figure 9:**
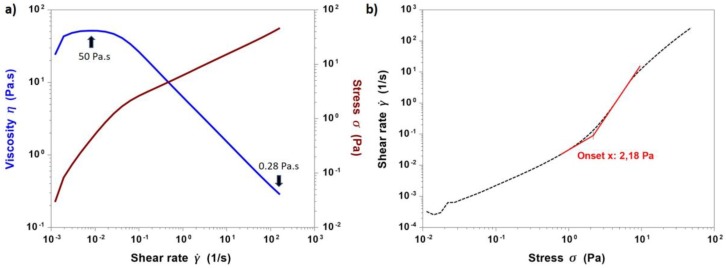
Flow ramps performed on collagen/MBG_Sr4% suspensions at 4 °C showing viscosity and stress variation with increasing shear rate values (**a**) and the shear rate pattern as a function of stress variation (**b**).

The shear stress-induced change in the material viscosity was further investigated by performing flow ramps at 4 °C. In Figure 9a, viscosity and stress values were plotted as a function of a wide range of increasing shear rates (10^−3^–200 s^−1^), while Figure 9b reported the shear rate pattern as a function of stress variation (0.01–60 Pa).

The resulting data proved the pseudo-plasticity (*shear thinning*) of collagen/MBG suspensions given by the rapid decrease of the material viscosity with increasing shear rates, starting from about 50 Pa·s at 0.01 s^−1^ and reaching values of about 0.28 Pa·s at 200 s^−1^. In addition, a yield stress value of 2.18 Pa was detected by measuring the onset point corresponding to the curve slope change in the second graph (Figure 9b). In particular, collagen was widely reported to show shear thinning flow behaviour due to the polymeric chain orientation according to the applied stress [28,30,48]. Therefore, rheological studies have proven that the addition of MBG_Sr4% particles did not prevent the molecular reorganisation, while the presence of yield stress further increased the material stability upon deposition [28,29].

With the aim to better improve the stability after extrusion, materials performing sol-gel transition in response to physical and chemical stimuli are considered among the most eligible for 3D printing applications [28,29]. The gelation kinetics of collagen/MBG suspensions were, thus, investigated observing the variation of G’ (storage modulus) and G’’ (loss modulus) at 37 °C for up to 1 h.

**Figure 10:**
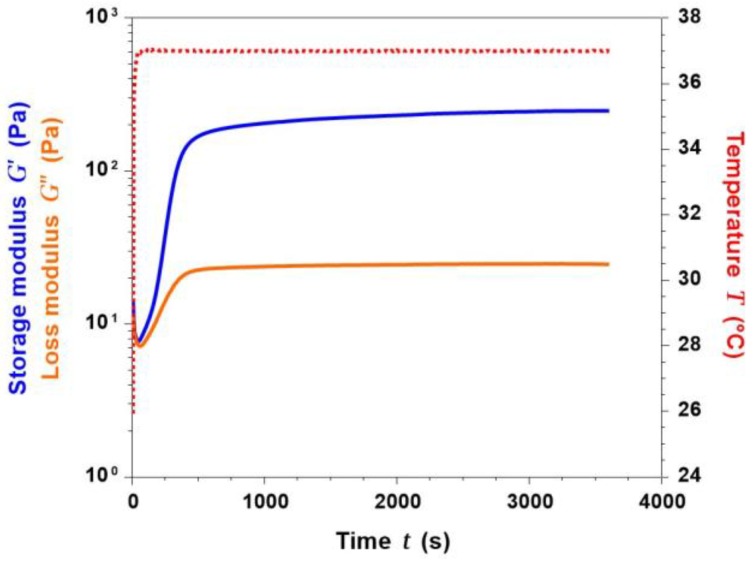
Sol-gel transition of collagen/MBG_Sr4% systems detected through the time sweep test at 37 °C.

As reported in Figure 10, the sol-gel transition of collagen/MBG suspensions, identified by the sharp increase of G’ compared to G’’, was detected after less than 50 seconds. In addition, a stable gel was formed after about 8 min, detecting a significant gap between G’ and G’’. Greater values of G’ compared to G’’ were indicative of a major elastic response over the viscous behaviour [10]. As a result of the collagen fibre reconstitution, increasingly-stronger gels were obtained after up to 1 h at 37 °C, reporting a final G’ value of 248.16 Pa. The rapid gel formation induced by a constant temperature of 37 °C can, therefore, be exploited to prevent the material from spreading out after the 3D structure deposition.

Although the reported rheological properties are promising, the low value of yield stress and the short time gap required to form a stable gel may prevent the immediate stabilisation of the material upon deposition, leading to a limited printing fidelity [31,32]. In order to address this crucial aspect, future studies will be focused on the improvement of the material properties, by considering both higher collagen concentrations and the use of alternative inorganic phases.

#### 3.5.2. Analyses on Collagen/MBG_Sr4% Composites

As previously reported, the fibril reconstitution, and the consequent sol-gel transition of collagen-based materials, is known to be affected by different parameters such as pH, ionic concentration, as well as incubation temperature and time [14].

The influence of the increased time of incubation at 37 °C on the visco-elastic properties of collagen/MBG constructs was studied by performing amplitude sweep tests on gel samples. In detail, the collagenous suspension was allowed to gel and kept at 37 °C for 3 h before starting the analysis. Subsequently, G’ and G’’ values were measured varying the oscillation strain until a linear visco-elastic behaviour was detected.

**Figure 11:**
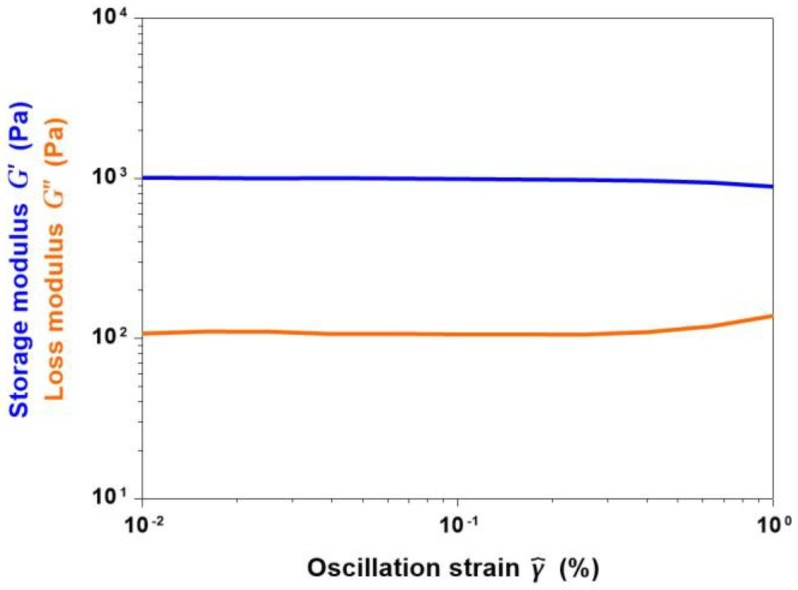
Amplitude sweep test showing G’ (storage modulus) and G’’ (loss modulus) values of collagen/MBG_Sr4% samples after 3 h of incubation at 37 °C.

**Figure A4:**
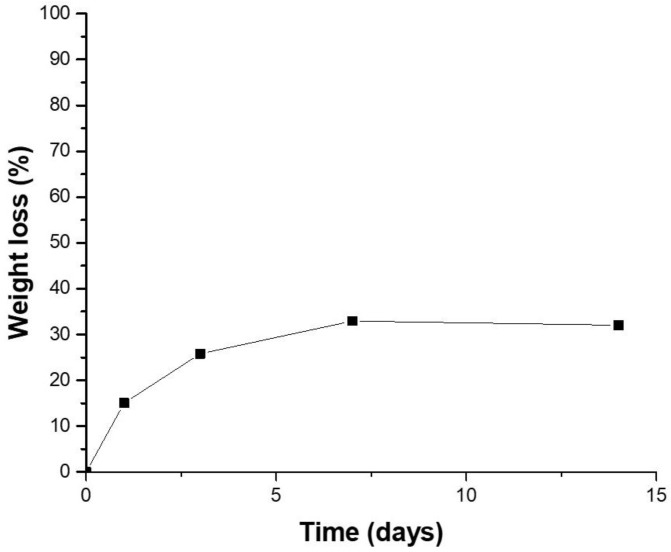
Percentage of weight loss of collagen/MBG_Sr4% samples incubated in PBS at 37 °C up to 14 days. The weight loss percentage has been calculated on samples collected at different time points (1, 3, 7, 14 days) and subsequently lyophilised. The graph shows that the material is able to maintain more that 60% of its initial weight up to 14 days of incubation.

Figure 11 reported the pattern of G’ and G’’ for oscillation strains ranging from 0.01% to 1%. The large gap detected between the storage and loss moduli further confirmed the solid nature of the samples. The averaged values of G’ and G’’ in the considered region was measured to be about 950 Pa and 123 Pa, respectively. Compared to values collected after 1 h, a significant increase of the visco-elastic modulus was detected after 3 h at 37 °C. The resulting data suggested the relevant influence of higher gelation times on the final mechanical properties of collagenous composites, in accordance with the data found in the literature [8]. Collagen/MBG_Sr4% samples were, in fact, able to maintain more than 60% of their initial weight after 14 days of incubation in PBS at 37 °C (Figure A4). However, the reported values of complex modulus are still far from those reported for hard tissues, suggesting the need to enhance the visco-elastic properties of the material after gelation [34]. The combination of collagen with additional inorganic particles, higher collagen concentrations, and the employ of a suitable crosslinking method will be considered as future steps to better improve the final properties of the composite and the overall material stability.

### 3.6. Preliminary Extrusion Tests with 32 G Needles

**Figure 12:**
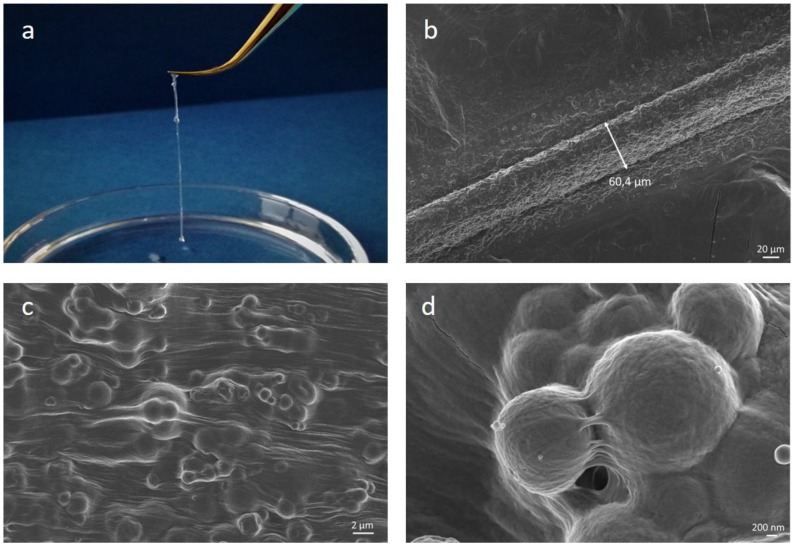
Extrusion tests on collagen/MBG_Sr4% hybrid systems. Images show the extruded filament immediately after deposition (**a**) and FESEM analyses on lyophilized samples reproducing the filament diameter (**b**) and nanostructure at different magnifications (**c**,**d**).

Preliminary extrusion tests were conducted on collagen/MBG suspensions using a syringe equipped with 32 G needles in order to investigate their suitability for the design of high-resolution structures. As shown in Figure 12, the collagenous suspension was successfully extruded creating solid strands of about 60 μm once deposited on surfaces pre-heated at 37 °C in order to promote the physical crosslinking of collagen. FESEM images of collected filaments clearly showed the collagen fibre reconstitution and the proper distribution and embedding of the MBG inorganic particles, as previously reported for the nanostructural analysis of bulk samples.

In addition, the extrusion process led to the collagen fibre orientation along the shear flow direction, primarily due to the predominance of shear stresses on frictional forces existing between the randomly-arranged fibrils, as also suggested by Miri et al. [1]. Rheological analyses and preliminary extrusion tests have proven the suitability of the developed collagen-based hybrid suspensions for the design of structures by means of 3D extrusion printing using very thin needles (32 G).

## 4. Conclusions

This study aimed to develop a hybrid bioactive material suitable for the design of nanostructured scaffolds for bone tissue engineering by means of 3D printing technology. Strontium-containing MBG microspheres were homogeneously incorporated in the collagenous matrix without affecting the reconstitution of collagen fibrils. The composite material showed pronounced bioactive properties as a result of the fast and conspicuous HA crystal deposition confirmed by FESEM, ATR_FTIR, and XRD analyses. The great water uptake ability promoted the release of strontium ions within three days of incubation, proving that the collagenous matrix enabled the regular ion exchange between the MBG particles and the soaking medium. Rheological analyses highlighted the shear thinning behaviour and the yield stress of the collagenous suspensions, fundamental properties for printable biomaterials. The fast sol-gel transition triggered by a physiological temperature of 37 °C can further improve the material stability after extrusion. The results obtained have, thus, proven the potential of the proposed collagen/MBG_Sr4% hybrid system as promising bioactive materials for 3D printing of nanostructured 3D constructs mimicking the complex nature of bone tissue.

The presented results pave the way for further studies aiming at the manufacturing of biomimetic and bioactive 3D scaffolds by means of biofabrication platforms. The introduction of additional inorganic phases and the assessment of effective cross-linking methods to improve the overall properties of the final composite in terms of mechanical properties and stability in aqueous medium will be investigated.
